# Analysis of microorganisms and drug-resistance mutations detected by probe-capture metagenomics among HIV-infected patients with pneumonia

**DOI:** 10.3389/fmicb.2025.1616937

**Published:** 2025-07-28

**Authors:** Qianhui Chen, Pingzheng Mo, Rongrong Yang, Huan Xu, Huifang Liu, Zhongwei Zhang, Qian Du, Qunqun Jiang, Qinglian Guo, Liangjun Chen, Yongxi Zhang, Yong Xiong, Liping Deng

**Affiliations:** ^1^Department of Infectious Diseases, Zhongnan Hospital of Wuhan University, Wuhan, China; ^2^AIDS Research Center, Wuhan University, Wuhan, China; ^3^Vision Medicals Co. Ltd., Guangzhou, China; ^4^Department of Clinical Laboratory, Zhongnan Hospital of Wuhan University, Wuhan, China

**Keywords:** microorganisms, probe-capture metagenomics, HIV, pneumonia, drug resistance

## Abstract

**Background:**

Probe-Capture Metagenomics is a newly developed method for detecting infectious pathogens. However, its application in HIV-infected patients with pulmonary infection remains limited. This study utilized Probe-Capture Metagenomics to analyze lung microbiomes and Drug Resistance Mutations of HIV and bacteria in people living with HIV (PLWH) with pneumonia.

**Methods:**

We retrospectively investigated lung microorganisms in PLWH hospitalized at Zhongnan Hospital of Wuhan University. A combination of bronchoalveolar lavage fluid Probe-Capture Metagenomics and conventional microbiological tests were performed in all patients.

**Results:**

A total of 91 patients were included in the study. Excluding the EB and Torque teno virus, at least two organisms were identified in 85 patients using Probe-Capture Metagenomics combined with conventional microbiological tests. The top six detected organisms were CMV, *Pneumocystis jirovecii*, *Mycobacterium tuberculosis complex*, HHV-7, *Candida albicans and Aspergillus*. For specific organisms, the detection rate of CMV and *Candida albicans* by Probe-Capture Metagenomics was significantly higher than that of conventional microbiological tests (*p* < 0.0001). Moreover, the detection rates of CMV (*p* = 0.0167) and *Pneumocystis jirovecii* (*p* = 0.04) in patients with CD4^+^T count ≥ 200 cells/μL were higher than that with CD4^+^T count < 200 cells/μL. Importantly, Probe-Capture Metagenomics can uncover potentially clinically relevant drug-resistance mutations linked to HIV and bacteria.

**Conclusion:**

Probe-Capture Metagenomics provides a promising method of detecting suspected opportunistic infections in PLWH with pneumonia, especially for mixed infections and rare microorganisms. In addition, Probe-Capture Metagenomics was a potential valuable tool for genotyping resistance testing of HIV and bacteria.

## 1 Introduction

Lower respiratory tract infections can be caused by a variety of microbes including bacteria, fungi and viruses, which was one of the main causes of morbidity and mortality among people living with HIV (PLWH), especially in low-income countries countries (Zarrinfar et al., 2012; Zarrinfar et al., 2014; Zarrinfar et al., 2016). Pneumonia diagnoses in PLWH with immune dysfunction pose an additional challenge as these patients are susceptible to mixed respiratory pathogens (Di Pasquale et al., 2019; Wang et al., 2019). Patients with mixed pulmonary infection may exhibit a more severe and diverse clinical manifestations compared to those with a single pathogen infection. In addition, conventional microbiological tests (CMTs) of detecting microorganisms have several limitations, including low positivity rates, long culture times, susceptibility to interference from empirical antibiotic treatment, and difficulties in detecting uncommon or emerging pathogens (Xu et al., 2022). Each of these causes contributes to the complexity of the diagnosis. Therefore, timely and precise identification of multi-pathogenic pneumonia is crucial for optimizing treatment strategies, improving the prognosis, and reducing mortality rates.

In recent years, metagenomic high-throughputhigh-throughput sequencing (mHTS) has been around for over two decades now (since the onset of the 2nd gen sequencing with 454), gradually gaining utility in clinical diagnosis expanded to the field of infectious diseases due to its rapid, unbiased and highly sensitive for identification of pathogens (Gu et al., 2019; Wüthrich et al., 2016; Shi et al., 2022). It can simultaneously detect various infectious pathogens, which provides additional valuable information for the detection of pathogens (Gu et al., 2019; Wüthrich et al., 2016; Shi et al., 2022). However, the large proportion of host DNA in human cells may mask other nucleic acids and affect the detection of certain microbial populations. DNA and RNA need to be processed separately, and the application of mHTS has mainly detected DNA microorganisms previously. Currently, RNA viruses, such as influenza virus, are also part of the important causes of lung infections. We developed a Probe-Capture Metagenomics assay for detecting infectious pathogens that integrates metagenomics techniques with magnetic bead capture technology (Sun et al., 2025). It employs a hybridization capture-based approach using pathogen-specific probes to enrich microbial genomic material while simultaneously depleting host DNA, and DNA/RNA library detection could be performed simultaneously. Thus, in addition to the advantages of mHTS, Probe-Capture Metagenomics can also detect RNA viruses and microbial resistance genes. However, the application of Probe-Capture Metagenomics in PLWH with pulmonary infection remains limited.

Therefore, the aim of this study was to explore the microbiome spectrum in PLWH with pneumonia using Probe-Capture Metagenomics, and to initially investigate the resistance gene phenotypes of respiratory pathogens and HIV in PLWH with pneumonia.

## 2 Materials and methods

### 2.1 Study design and participants

**Figure 1:**
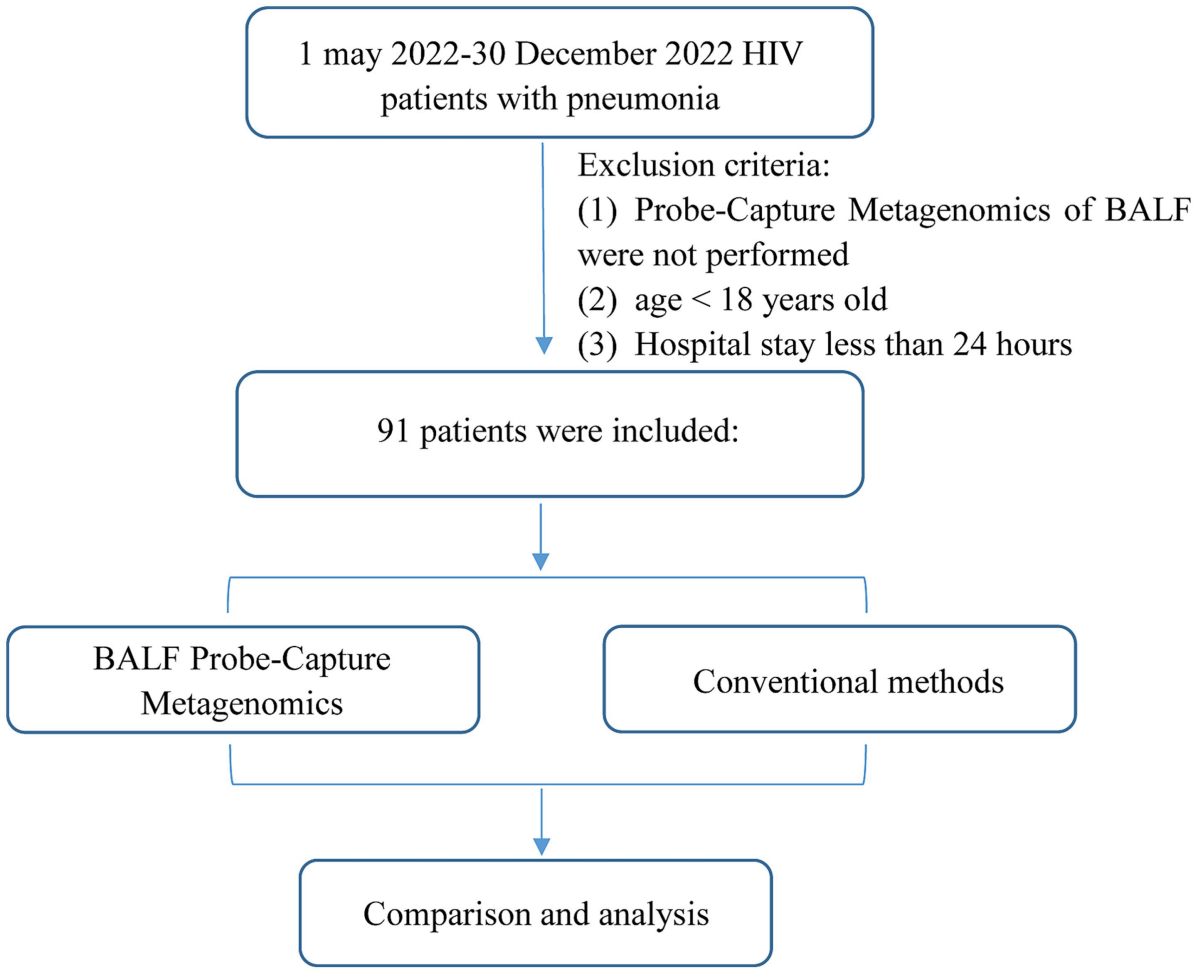
Flowchart of case selection. A total of 91 HIV-infected cases in the infection department were selected for further analysis.

Bronchoalveolar Lavage Fluid (BALF) samples from PLWH with pneumonia were submitted to Probe-Capture Metagenomics, which were retrospectively reviewed from the Zhongnan Hospital of Wuhan University from 1 may 2022 to 30 December 2022. Patients who met the following criteria were considered as pneumonia: (1) exhibited at least one of the following manifestations: fever; cough, expectoration, dyspnea or other respiratory symptoms; (2) chest imaging suggested pneumonia; (3) determined by the consensus of two experienced senior clinicians based on the clinical manifestation, laboratory tests, chest imaging and response to antimicrobial treatment. The exclusion criteria were as follows: (1) Probe-Capture Metagenomics of BALF were not performed; (2) age < 18 years old; (3) Hospital stay less than 24 h (Figure 1). At the same time, CMTs, including chest imaging, smear and culture of bacteria and fungi, 1,3-*β*-D glucan (G) assay (G test) (β-D-glucan detection kit, Shanghai Fuxing Changzheng Medical Science Co., Ltd., China) and galactomannan (GM) assay (Platelia^™^ Aspergillus Ag, Bio-Rad, United States), polymerase chain reaction (PCR) test [including cytomegalovirus (CMV), EB virus, *Pneumocystis jirovecii* (*P. jirovecii*), *Mycobacterium tuberculosis complex* (MTB)], tuberculin skin test (TST), interferon-gamma release assay (T-SPOT.TB kit, TB.300, Oxford Immunotec, UK), acid-fast stain (TB Auramine-Rhodamine kit, K12345, Thermo Fisher, United States) and GeneXpert MTB/RIF (Xpert) (Cepheid. *Xpert^®^ MTB/RIF Assay,* Cepheid, United States), were performed for pulmonary infection. The sequence and conditions of the primer pairs used for *P. jirovecii PCR* was described in one previous study (Chen et al., 2024). The primers for CMV PCR were as follows: Forward: 5’-CGCGACTTGACCTGGACTAC-3′, Reverse: 5’-TGGTGGCAGTGTACCCGTAA-3′. The conditions used were: 45 cycles of denaturation for 5 min at 95°C; annealing for 15 s at 95°C; and extension for 30 s at 60°C. The primers for EB PCR were as follows: Forward: 5’-GCCAGAGGTAAGTGGACTTT-3′, Reverse: 5’-TCTTGGTGAGCGGTAGTAAA-3′. The conditions used were: 40 cycles of denaturation for 3 min at 95°C; annealing for 10 s at 95°C; and extension for 30 s at 58°C. The primers for MTB PCR were as follows: Forward: 5’-CCTGCGAGCGTAGGCGTCGG-3′, Reverse: 5’-CTCGTCCAGCGCCGCTTCGG-3′. The conditions used were: 50 cycles of denaturation for 10 min at 95°C; annealing for 15 s at 95°C; and extension for 60 s at 62°C. The date of collection, symptoms and test results were included in the clinical data. All data was anonymized in our study. These patient clinical data was included as Supplementary Material. Every fungi/bacteria/virus evaluated in CMTs were present in the databases of Probe-Capture Metagenomics. The diagnosis was made based on clinical symptoms, laboratory findings, microbiologic examination, chest radiology, and the response to treatment. This study was approved by the institutional ethics committee of Zhongnan hospital of Wuhan university (2022101). All subjects have voluntarily taken part in the research and signed the informed consent form. The Declaration of Helsinki’s standards and any applicable laws were followed when conducting the study.

### 2.2 Data collection

Clinical data were retrospectively obtained from medical records. These following data were extracted: age, gender, HIV infection status, antiretroviral therapy (ART), antibiotic use within 3 months, lymphocyte count, CD4^+^ T lymphocyte count (CD4 count), HIV viral load, C-reactive protein (CRP), Interleukin-6 (IL-6), procalcitonin (PCT), erythrocyte sedimentation rate (ESR), D-dimer, lactate dehydrogenase (LDH), alanine aminotransferase (ALT), albumin, creatinine, results of CMTs, chest imaging, and Probe-Capture Metagenomics tests.

### 2.3 Probe design for pathogen detection

To optimize probe design, we employed HUBDesign, a bioinformatics pipeline that generates probes from representative sequences at multiple taxonomic levels. This method allows for precise targeting and enrichment of specific clades, facilitating the detection of both known and novel organisms. The default parameters of HUBDesign were applied in this study. We designed a comprehensive probe library targeting up to 167 clinically relevant pathogenic microorganisms. And the pathogen-specific probe set includes 62 bacteria and 41 antimicrobial resistance genes, 16 fungi, 14 parasites, 15 DNA viruses, and 60 RNA viruses, collectively covering >95% of pathogens detected in clinical samples. A total of 640 probes were designed including 517 probes targeting pathogens and 183 probes targeting resistance genes. Pathogens used for probe design are provided in Supplementary Materials. Leveraging sequence homology and DNA hybridization, this pipeline has been successfully demonstrated in designing and validating probe sets for two key applications: (1) comprehensive detection of all sequenced coronaviruses, and (2) identification of bacterial pathogens associated with sepsis. The Probe-Capture assay developed in this work has been granted an invention patent by the China National Intellectual Property Administration (patent name: Group, method, kit and application of Probe Capture for detecting pathogenic microorganisms, patent No. ZL2020116367800).

### 2.4 Nucleic acid extraction, library preparation, probe hybridization and sequencing

DNA was extracted from all samples using a QIAamp^®^ UCP Pathogen DNA Kit (Qiagen) following the manufacturer’s instructions. Human DNA was removed using Benzonase (Qiagen) and Tween20 (Sigma). Using a Qubit dsDNA HS Assay Kit (Invitrogen, United States), the isolated DNA was quantified. Total RNA was extracted with a QIAamp^®^ Viral RNA Kit (Qiagen) and ribosomal RNA was removed by a Ribo-Zero rRNA Removal Kit (Illumina). cDNA was generated using reverse transcriptase and dNTPs (Thermo Fisher). Libraries were constructed for the DNA and cDNA samples using a Nextera XT DNA Library Prep Kit (Illumina, San Diego, CA). An aliquot of 750-ng library from each sample was used for hybrid capture-based enrichment of microbial probe one rounds of hybridization (SeqCap EZ Library, Roche, United States). Purification and size selection were carried out following the double-sided bead purification procedure. Library was quality assessed by Qubit dsDNA HS Assay kit followed by High Sensitivity DNA kit (Agilent) on an Agilent 2,100 Bioanalyzer. Library pools were then loaded onto an Illumina Nextseq CN500 sequencer for 75 cycles of single-end sequencing to generate approximately 20 million reads for each library. For negative controls, we also prepared sterile deionized water in parallel with each batch to serve as non-template controls (NTC), using the same protocol.

### 2.5 Bioinformatics analyses

Raw reads were adapter trimmed and quality filtered using Trimmomatic (v0.39). All reads longer than 40 base pairs with a minimum phred quality score of 30 were kept for further analysis (Bolger et al., 2014). Low-complexity reads were removed by fastp (v0.23.4) default settings (Chen et al., 2018). The human sequence data were identified and eliminated by mapping them to the hg38 reference genome utilizing Burrows-Wheeler Aligner software (BWA-0.7.17) (Li and Durbin, 2009). Taxonomic classification of sequencing reads was performed using Kraken2 (v2.1.3) with default parameters, including a confidence threshold of 0.2 (-confidence 0.2) and 40 threads (-threads 40). The Kraken2 reference database was constructed using the kraken2-build –standard pipeline, incorporating complete genomes and assemblies from the NCBI RefSeq archaea, bacteria, viruses, plasmids, human (GRCh38), UniVec_Core, protozoa, fungi and plants, all downloaded on December 28, 2024. To enhance microbial representation, genomes of Pneumocystis jirovecii (GenBank accession no. GCA_001477535.1) were manually incorporated into the database, as detailed in Supplementary Table 1. To mitigate spurious alignments arising from PCR duplicates or low complexity regions, microbial reads were subsequently aligned against the same reference database using the Burrows–Wheeler Aligner (BWA, v0.7.17) with default parameters. Only alignments exhibiting a nucleotide identity of ≥96% to the reference genome were considered valid. Furthermore, reads aligning to multiple loci within the same genus were excluded from downstream analyses to prevent ambiguous taxonomic assignments. To remove the mistakes brought on by different sequencing depths between samples, we normalized the sequencing reads using reads per million (RPM). The clinical reportable range (CRR) for pathogens was established according to the following three references indicated in a previous study (Jing et al., 2021): I. Johns Hopkins ABX Guide,^1^ II. Manual of Clinical Microbiology, and III. clinical case reports or research articles published in peer-reviewed journals.

The clinical reportable range (CRR) for pathogens was established according to a previous study (Sun et al., 2025). The suspected pathogens were determined according to the following rules (Wilson et al., 2019): A virus was considered positively detected if RPM ≥ 3 covered at least three non-overlapping regions in the genome. For identification of bacteria, fungi, and parasites, we developed a RPM ratio metric, defined as RPM-r = RPM sample/RPM_NTC_, or RPM ratio = RPM sample if RPM_NTC_ = 0. A positive detection of a bacterium, fungus, or parasite required an RPM ratio ≥10. The results can be considered positive when they are consistent with the pathogenicity of positive microorganisms, clinical characteristics, and therapeutic efficacy.

### 2.6 Drug resistance mutations analysis

The Stanford HIV Genotypic Resistance Interpretation Algorithm^2^ was used to identify mutations associated with HIV drug resistance. HIVDR mutations linked with integrase strand transfer inhibitor (INSTI), reverse-transcriptase inhibitor (RT), and protease inhibitor (PI) were reported with threshold of mutation detection threshold (MDT) ≥ 1%, and divided into three categories: major, accessory, and other. The estimated drug resistance based on the total point score was designated: susceptible, potential low-level resistance, low-level resistance, intermediate resistance, and high-level resistance.

To identify clinically important antimicrobial resistance bacteria, such as carbapenem-resistant *Enterobacterales*, carbapenem-resistant *Acinetobacter baumannii*, carbapenem-resistant *Pesudomonas aerugeinosa*, third-generation cephalosporin-resistant *Enterobacterales*, extended-spectrum *β*-lactamases (ESBL) producing Enterobacteriaceae, vancomycin-resistant *Enterococcus faecium*, methicillin-resistant *Staphylococcus aureus*, and so on, the specimen’s sequence was screened and aligned with sequences of antibiotic resistance genes in the Comprehensive Antibiotic Resistance Database (CARD; https://card.mcmaster.ca) (Jia et al., 2017). This analysis was only performed when the targeted bacterial sequence surpassed a threshold of 1,000. And the relationship between antibiotic resistance genes and the detected bacteria was evaluated using the CARD database (Alcock et al., 2023).

### 2.7 Statistical analysis

SPSS 25.0 software (IBM Corp., Armonk, NY, United States) was used for statistical analysis. The numerical variables were presented as medians and interquartile ranges and nominal variables as counts and percentages. Comprehensive clinical diagnosis and determination of microbial aetiology were used as reference standards. The McNemar test was used to compare the diagnostic performance of CMTs and Probe-Capture Metagenomics. Fisher’s exact test was used to test for association of pathogen with the categorical variables of CD4 count (≥200 vs. < 200 cells per μL). All tests were two-tailed, and the *p* value of <0.05 was considered statistically significant.

## 3 Results

### 3.1 Patient characteristics

During the study period, a total of 91 HIV patients (mean age: 48 years) including 78 men (85.7%) met the inclusion criteria. Upon admission to hospital, the median CD4 count of 91 patients was 57 cells/μL. 48 (52.7%) were taking antiretroviral therapy, and 16 (17.58%) had been receiving *P. jirovecii* pneumonia prophylaxis. 10 (11.24%) out of 89 patients were receiving empirical antifungal therapy, and 62 (68.13%) out of 91 patients were receiving empirical antibiotic therapy. 57 patients (62.64%) had fever, and 72 patients (79.12%) of CT scan of the lung were abnormal. Detailed clinical characteristics are shown in Table 1.

**Table 1:** Clinical and laboratory characteristics of 91 PLWH with pulmonary infection.

Characteristics	Mean ± SD/number (%)/median (IQR)	Total number of patients assessed
Total		91
Age	48.0 ± 13.3	91
Male	78 (85.7)	91
Past medical history		91
Hypertension	8 (8.8)	
Diabetes	5 (5.5)	
Tuberculosis	8 (8.8)	
Hepatitis	9 (9.9)	
Syphilis	4 (4.4)	
Cardiovascular and cerebrovascular diseases	5 (5.5)	
Chronic airway diseases	2 (2.2)	
Tumor	4 (4.4)	
Receiving antiretroviral therapy	48 (52.7)	91
Receiving *Pneumocystis jirovecii* prophylaxis	16 (17.58)	91
Receiving antifungal therapy	10 (11.24)	89
Background antibiotic use	62 (68.13)	91
Any antibiotic	28 (31.46)	
Ceftriaxone	11 (12.36)	
Quinolone	6 (6.74)	
Penicillin	5 (5.62)	
Carbapenems	5 (5.62)	
β-lactam/β-lactamase inhibitors	4 (4.49)	
Glycopeptide	3 (3.37)	
Symptoms and signs		91
Fever	57 (62.64)	
Cough	65 (71.43)	
Chest tightness	38 (41.76)	
Shortness of breath	35 (38.46)	
Fatigue	14 (15.38)	
Days from illness onset to first admission	30 (15, 60)	91
Days of hospital stay	13.86 ± 7.29	91
LgHIV-VL	0 (0, 10,900)	69
Abnormal CT scan of lung	72 (79.12)	91
Blood routine and biochemistry results		91
White blood cell count, × 10^9^/L	4.77 ± 2.50	
Hemoglobin, g/L	100.76 ± 20.60	
Platelet count, × 10^9^/L	211.90 ± 103.2	
Alanine aminotransferase, U/L	20.5 (12.75, 38.25)	
Albumin, g/L	30.4 (27.08, 34.82)	
Creatinine, μmol/L	64.6 (59.12, 79.58)	
CD3^+^ CD4^+^T cells	57 (14, 120)	91
Lactate dehydrogenase, U/L	279.5 (174.5,382.25)	60
D-dimer, ng/mL	448 (217.25, 949.75)	88
Infection-related biomarkers		
ESR, mm/h	63.79 ± 35.75	80
C-reactive protein, mg/L	33.4 (16.05, 88.65)	60
Interleukin-6, pg./mL	26.35 (14.2, 62.1)	60
Procalcitonin, ng/mL	0.01 (0.01, 0.39)	91

### 3.2 Distribution of pathogens

**Figure 2:**
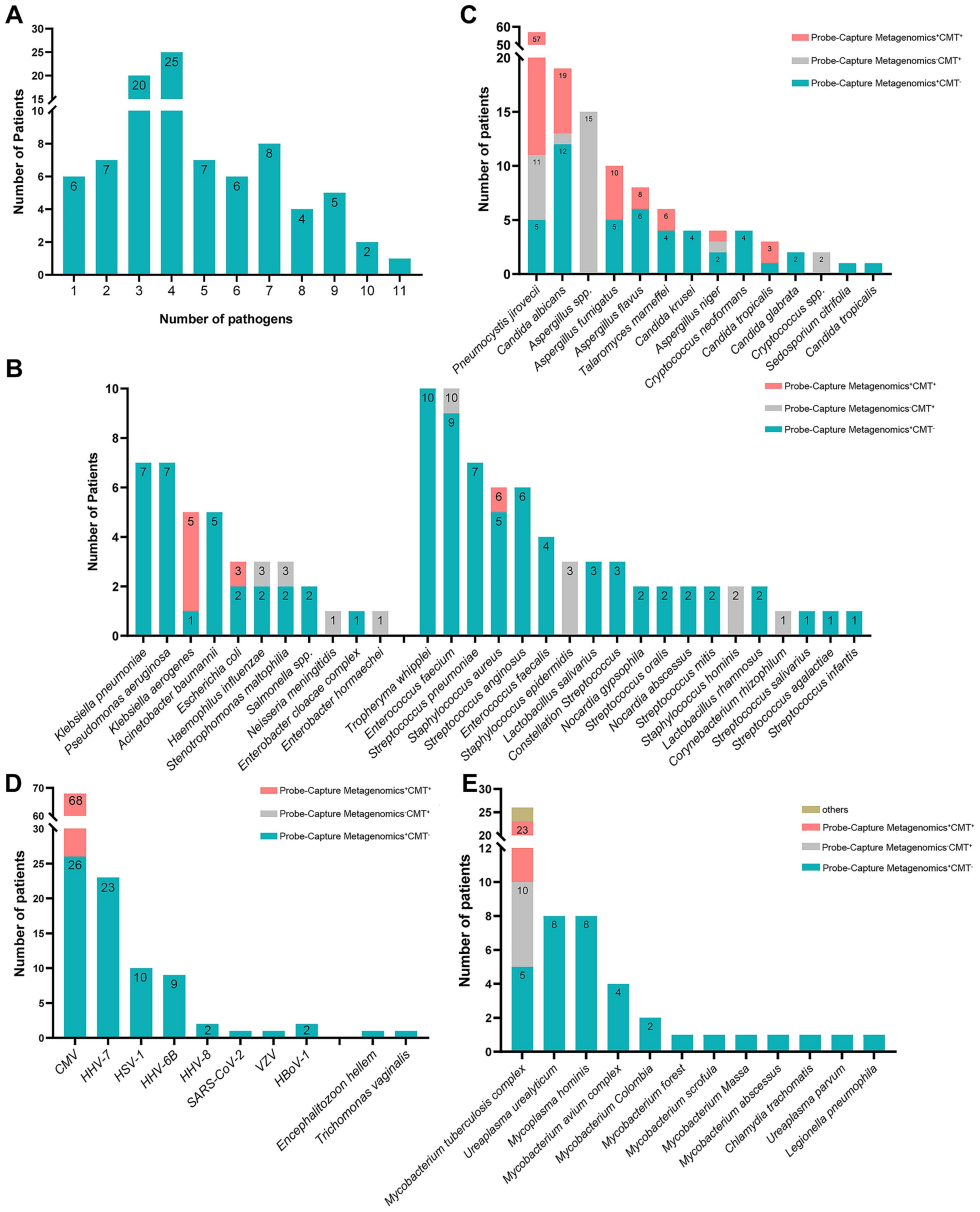
The organism spectrums of pulmonary detected by probe-capture metagenomics and CMTs in PLWH with pneumonia. **(A)** number of patients in different amounts of detected organisms; **(B)** number of patients in different amounts of detected bacterial species; **(C)** number of patients in different amounts of detected fungus species; **(D)** number of patients in different amounts of detected virus species; **(E)** number of patients in different amounts of detected other atypical pathogen species. PLWH, people living with HIV; CMTs, conventional microbiological tests; CMV, cytomegalovirus; HHV-7, human herpes virus 7; HSV-1, herpes simplex virus 1; HHV-6B, human herpes virus 6B; HHV-8, human herpes virus 8; VZV, varicella-zoster virus; HBoV-1, human bocavirus 1.

Of 91 samples, excluding the EB and Torque teno virus, 6 had single pathogen and 85 had at least two microorganism (Figure 2A) detected by Probe-Capture Metagenomics combined with CMTs. In addition, the top six organisms were CMV, *P. jirovecii*, MTB, HHV-7, *Candida albicans* (*C. albicans*) *and Aspergillus* (Figures 2B–E).

As shown in Figure 2B, *Tropheryma whipplei (T. whipplei)*, *Enterococcus faecium*, *Klebsiella pneumoniae*, *Pseudomonas aeruginosa* and *Streptococcus pneumoniae* were more prevalent in the detected bacteria spectrum among PLWH with pulmonary infection.

As shown in Figure 2C, *P. jirovecii* and *C. albicans* were more common in the detected fungus spectrum. Among the detected fungus, two kinds (*Aspergillus* and *Cryptococcus*) of them were detected by CMTs only, five kinds (*Candida krusei*, *Cryptococcus neoformans*, *Candida glabrata*, *Sedosporium citrifolia* and *Candida tropicalis*) of them were detected by Probe-Capture Metagenomics only. In the cases of *P. jirovecii* detection, 5 cases were diagnosed by Probe-Capture Metagenomics alone, and 6 cases were diagnosed by CMTs alone. In the cases of *C. albicans* detection, 12 cases were diagnosed by Probe-Capture Metagenomics alone, and 1 case was diagnosed by CMTs alone. In the cases of *Aspergillus fumigatus* detection, 5 cases were diagnosed by Probe-Capture Metagenomics alone. In the cases of *Aspergillus flavus* detection, 6 cases were diagnosed by Probe-Capture Metagenomics alone and 2 cases were diagnosed both by Probe-Capture Metagenomics and CMTs. In the cases of *T. marneffei* detection, 4 cases were diagnosed by Probe-Capture Metagenomics alone. In the cases of *Aspergillus niger* detection, 2 cases were diagnosed by Probe-Capture Metagenomics alone, and 1 case was diagnosed by CMTs alone. In the cases of *Candida tropicalis* detection, 1 case was diagnosed by Probe-Capture Metagenomics alone.

As shown in Figure 2D, CMV and HHV-7 were more common in the detected virus spectrum. In the cases of CMV detection, 26 case was diagnosed by Probe-Capture Metagenomics alone. In addition, except for CMV, the remaining viruses were detected only by Probe-Capture Metagenomics.

As shown in Figure 2E, MTB and *Mycobacterium avium* complex (MAC) were more common in the detected mycobacterium spectrum. In the cases of MTB detection, 5 case was diagnosed by Probe-Capture Metagenomics alone, 13 cases were diagnosed both by Probe-Capture Metagenomics and CMTs, and 5 cases were diagnosed by CMTs alone. In addition, all of the nontuberculous mycobacteria (NTM), *Mycoplasma hominis*, *Chlamydia trachomatis*, Ureaplasma and *Legionella pneumophila* were detected only by Probe-Capture Metagenomics.

### 3.3 Comparison of diagnostic performance among probe-capture metagenomics and CMTs in PLWH with pneumonia

As shown in Table 2 and Supplementary Table 2, Probe-Capture Metagenomics demonstrated superior detection performance compared to CMTs for CMV and *C. albicans* infections in PLWH with pulmonary infections. Using the McNemar test to compare paired binary outcomes (positive/negative) from Probe-Capture Metagenomics and CMTs, Probe-Capture Metagenomics showed significantly higher detection rates for CMV (*p* < 0.001) and *C. albicans* (p < 0.001). For CMV viremia, Probe-Capture Metagenomics achieved a sensitivity of 100% (95% CI, 94.65–100%) compared to 67.65% for CMTs (95% CI, 55.84–77.56). Similarly, for *C. albicans* infection, Probe-Capture Metagenomics sensitivity was 94.74% (95% CI, 75.36–99.73), significantly higher than CMTs at 31.58% (95% CI, 15.36–53.99; *p* < 0.001). In contrast, BALF Probe-Capture Metagenomics showed comparable detection performance to CMTs for *P. jirovecii*, MTB, and *Aspergillus* infections (*p* > 0.05, McNemar test).

**Table 2:** Comparison of diagnostic performance among probe-capture metagenomics and CMTs in PLWH with pulmonary infection.

	Probe-capture metagenomics		CMTs		*P* value
Pathogen	Sensitivity% (95% CI)	Specificity% (95% CI)	Sensitivity% (95% CI)	Specificity% (95% CI)	
CMV	100 (94.65–100)	100 (85.69–100)	67.65 (55.84–77.56)	91.3 (73.2–98.45)	<0.0001
*pneumocystis jirovecii*	84.48 (73.07–91.62)	100 (89.57–100)	84.48 (73.07–91.62)	100 (89.57–100)	1
MTB	65.38 (46.22–80.59)	100 (94.42–100)	69.23 (50.01–83.5)	96.92 (89.46–99.45)	0.548828
*Candida albicans*	94.74 (75.36–99.73)	83.33 (73.09–90.2)	31.58 (15.36–53.99)	100 (94.93–100)	<0.0001
*Aspergillus*	52.94 (36.74–68.55)	96.49 (88.08–99.38)	67.65 (50.84–80.87)	96.49 (88.08–99.38)	0.47313

### 3.4 Comparison analysis of lung microorganisms in patients with CD4^+^T count < 200 cells/μL and those with a count ≥ 200 cells/μL

We further compared the lung microorganisms of PLWH with pulmonary infection between patients with CD4^+^T count < 200 cells/μL to those with a count ≥ 200 cells/μL. As shown in Table 3, the detection ratios of CMV and *P. jirovecii* in patients with CD4^+^T count < 200 cells/μL were significantly higher than that of CD4^+^T count ≥ 200 cells/μL (*p* < 0.05). However, there are no significant differences of the detection ratios of MTB, *C. albicans*, NTM, *Aspergillus*, *T. marneffei*, HHV-7 and EBV between patients with CD4^+^T count < 200 cells/μL and CD4^+^T count ≥ 200 cells/μL (*p* > 0.05).

**Table 3:** Data and statistical calculations for CD4 count and pathogen detection.

Pathogen	CD4 < 200 cells/ul	CD4 ≥ 200 cells/ul	Fisher *p*	Adjusted *p*
CMV	59	9	0.013322	0.016746
No CMV	14	9		
*P. jirovecii*	50	7	0.029122	0.040034
No *P. jirovecii*	23	11		
*Mycobacterium tuberculosis* complex	20	6	0.771346	0.835192
No *Mycobacterium tuberculosis* complex	53	12		
*Candida albicans*	17	2	0.343282	0.415257
No *Candida albicans*	56	16		
Non-tuberculous Mycobacteria	9	1	0.679994	0.687518
No non-tuberculous Mycobacteria	64	17		
Aspergillus	28	6	0.789887	0.902466
No Aspergillus	45	12		
Talaromyces marneffei	6	0	0.594388	0.466422
No Talaromyces marneffei	67	18		
HHV-7	19	5	1	1
No HHV-7	54	13		

*p* values determined by Fisher’s exact test. Adjusted *p* values based on Benjamini-Hochberg multiple test correction.

### 3.5 The drug-resistance mutations (DRMs) associated with HIV and bacteria detected by probe-capture metagenomics in PLWH with pneumonia

**Figure 3:**
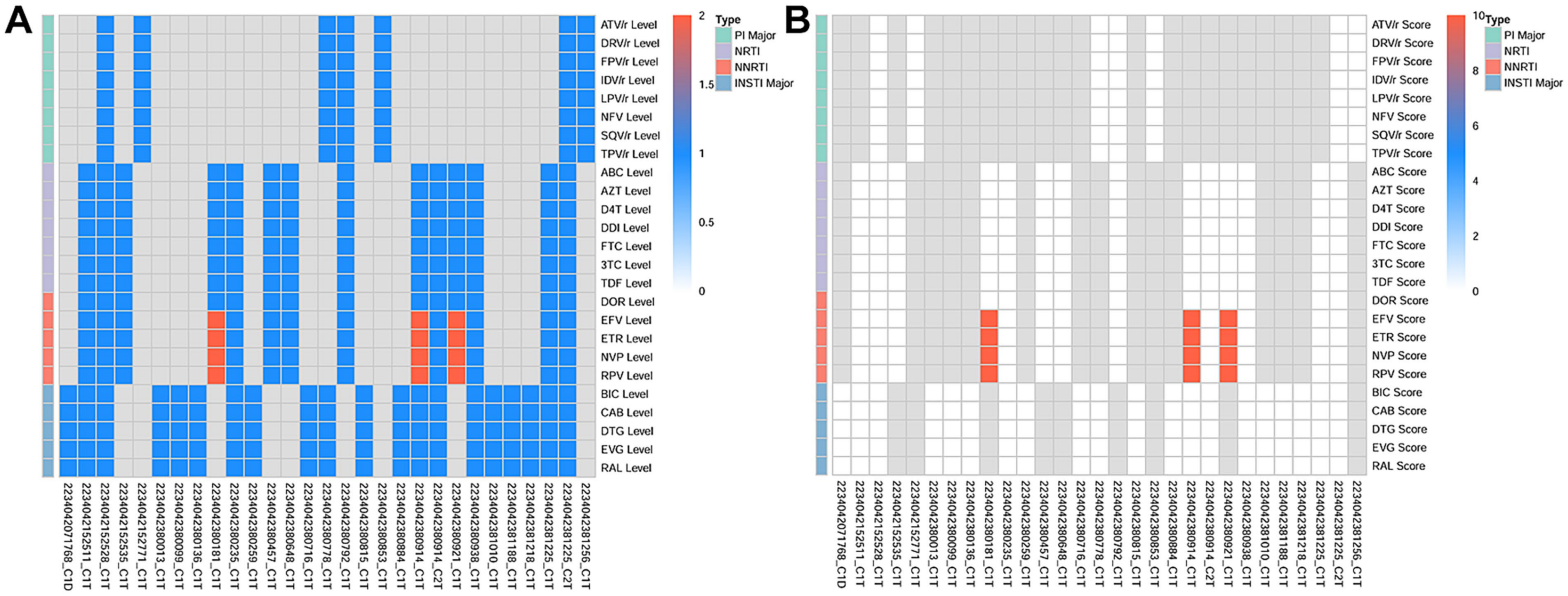
The DRMs associated with HIV detected in BALF by probe-capture metagenomics in PLWH with pneumonia. **(A)** The HIV resistance level. 1 represents susceptible. 2 represents potential low-level resistance. **(B)** The HIV resistance score of drug resistance analysis. A score of 0–9 is susceptible and a score of 10–14 is potential low-level resistance. The gray color indicates that no detection sequence is covered. DRMs, drug-resistance mutations; BALF, Bronchoalveolar Lavage Fluid; PLWH, people living with HIV; PIs, protease inhibitors; NRTI, nucleotide reverse transcriptase inhibitor; NNRTI, non-nucleoside reverse transcriptase inhibitors; INSTI, integrase inhibitor; ATV/r, Atazanavir/Ritonavir; DRV/r, Darunavir/Ritonavir; FPV/r, Fosamprenavir/Ritonavir; IDV/r, Indinavir/Ritonavir; LPV/r, Lopinavir/Ritonavir; NFV/r, Nelfinavir/Ritonavir; SQV/r, Saquinavir/Ritonavir; TPV/r, Tipranavir/Ritonavir; ABC, Abacavir; AZT, Zidovudine; D4T, Stavudine; DDI, Didanosine; FTC, Emtricitabine; 3TC, Lamivudine; TDF, Tenofovir Disoproxil Fumarate; DOR, Doravirine; EFV, Efavirenz; ETR, Etravirine; NVP, Nevirapine; RPV, Rilpivirine; BIC, Bictegravir; CAB, Cabotegravir; DTG, Dolutegravir; EVG, Elvitegravir; RAL, Raltegravir.

Furthermore, 91 samples were successfully sequenced to detect DRMs using Probe-Capture Metagenomics. HIV sequences were detected in 26 BALF samples. Detailed results are presented in the Supplementary Materials. As shown in Figure 3, DRMs (V179E, V179D) for nonnucleoside reverse transcriptase inhibitors (NNRTI) were found in the BALF of 3 patients. The scores of DRMs associated with HIV resistance were all 10 in each cases, indicating that HIV in these three patients had potentially low-level resistance to NNRTI (Figure 3B). In addition, the BALF of 27 patients exhibited the presence of antimicrobial resistance genes (ErmB, blaTEM, ErmC, mecA, blaCTX-M, blaSHV), which were identified by Probe-Capture Metagenomics (Table 4).

**Table 4:** Lower respiratory tract antimicrobial resistance genes identified by probe-capture metagenomics in 27 patients.

Test ID	Gene	Specific reads (*n*)	Class	Suspected associated bacteria
2234042380013	ErmB	350	Macrolide, Lincosomide	*Streptococcus pneumoniae*
2234042380198	blaTEM	178	Penicillinm, Cephalothin	*Haemophilus influenzae*
2234042380228	blaTEM	11	Penicillinm, Cephalothin	*Klebsiella pneumoniae*
2234042380259	ErmB	28	Macrolide, Lincosomide	*Staphylococcus aureus*
2234042380259	ErmC	1	Macrolide, Lincosomide	*Staphylococcus aureus*
2234042380259	mecA	4	Oxazocilline, Methicillin	*Staphylococcus aureus*
2234042380273	blaCTX-M	6	Penicillinm, Cephalothin	*Klebsiella pneumoniae*
2234042380273	blaSHV	11	Penicillinm, Cephalothin	*Klebsiella pneumoniae*
2234042380273	blaTEM	17	Penicillinm, Cephalothin	*Klebsiella pneumoniae*
2234042380297	ErmC	7	Macrolide, Lincosomide	*Staphylococcus aureus*
2234042380297	blaSHV	14	Penicillinm, Cephalothin	*Klebsiella pneumoniae*
2234042380341	blaCTX-M	214	Penicillinm, Cephalothin	*E. coli*
2234042380341	blaTEM	340	Penicillinm, Cephalothin	*E. coli*
2234042380358	ErmB	410	Macrolide, Lincosomide	*Streptococcus pneumoniae*
2234042380402	blaCTX-M	443	Penicillinm, Cephalothin	*E. coli*, *Klebsiella pneumoniae*
2234042380402	blaSHV	359	Penicillinm, Cephalothin	*E. coli*, *Klebsiella pneumoniae*
2234042380402	blaTEM	145	Penicillinm, Cephalothin	*Klebsiella pneumoniae*
2234042380457	ErmB	124	macrolide, lincosomide	*Streptococcus pneumoniae*
2234042380457	blaTEM	15	Penicillinm, Cephalothin	*Pseudomonas aeruginosa*
2234042380792	blaTEM	40	Penicillinm, Cephalothin	*Klebsiella pneumoniae*, baumannii
2234042380938	ErmB	3	Macrolide, Lincosomide	*Staphylococcus aureus*
2234042380938	mecA	5	Oxazocilline, Methicillin	*Staphylococcus aureus*
2234042381263	ErmB	178	Macrolide, Lincosomide	*Staphylococcus aureus*
2234042381263	ErmC	2	Macrolide, Lincosomide	*Staphylococcus aureus*
2234042381287	blaSHV	221	Penicillinm, Cephalothin	*Klebsiella pneumoniae*
2234042381287	blaTEM	34	Penicillinm, Cephalothin	*Haemophilus influenzae*
2234042381300	ErmB	18	Macrolide, Lincosomide	*Streptococcus pneumoniae*

## 4 Discussion

Various infections or frequent co-infections, including bacterial, fungal, viral and other atypical pathogens, are common in PLWH with severely immunosuppression (Chen et al., 2024). By utilizing Probe-Capture Metagenomics technology based on DNA/RNA library detection simultaneously, we facilitated the understanding of pneumonia microorganisms in the unique vulnerable population of PLWH. In addition to clinical manifestations and CMTs, the results of Probe-Capture Metagenomics can also provide potential reference for the clinical diagnosis and treatment of PLWH with pneumonia. Furthermore, its application in detecting drug-resistance mutations of HIV and bacteria was also initially explored for PLWH with pneumonia.

Currently, CMTs used in the clinic include the detection of specific antigens or antibodies, PCR, microscopy observation and culture. And culture and PCR in CMTs remain the primary detection methods, known as the gold standard for confirming microbial pathogen infection. However, there are certain limitations of CMTs, including low sensitivity, time-consuming procedures, difficulties in simultaneous detection of multiple pathogens (Jain et al., 2015; Labelle et al., 2010). In addition, there are numerous pathogens that cannot be effectively cultured or cultured for too long time within hospital settings (Wilson et al., 2014; Bateman et al., 2020; Sawatpanich et al., 2022; Getahun et al., 2007; Xie et al., 2018), such as *P. jirovecii*, NTM and MTB. Therefore, prompt appropriate diagnosis and treatment of pneumonia with co-infections in PLWH remains challenging. Probe-Capture Metagenomics is an emerging pathogen detection method based on molecular biology. This approach enables the targeting and enrichment of specific clades for the identification of both known and previously unidentified organisms. In our study, the Probe-Capture Metagenomics comprised a total of 640 probes, including 517 pathogen-specific probes and 183 antimicrobial resistance gene-targeting probes, which could cover >95% of pathogens detected in clinical samples. The top six detected organisms were CMV, *P. jirovecii*, MTB, HHV-7, *C. albicans and Aspergillus* using BALF Probe-Capture Metagenomics combined with CMTs. Furthermore, the sensitivity of Probe-Capture Metagenomics for the detection of CMV and *C. albicans* was markedly higher than that of CMTs. However, the sensitivity of BALF Probe-Capture Metagenomics was comparable to CMTs for diagnosing the infections of *P. jirovecii*, MTB, and *Aspergillus* in PLWH with pulmonary infection. While Probe-Capture Metagenomics offers technological advantages, its high cost currently limits practical application, especially in low- and middle-income countries. Therefore, the CMTs, such as PCR and Xpert, may be more appropriate for diagnosing *P. jirovecii*, *Aspergillus* and MTB in low- and middle-income areas. Furthermore, future development of additional CMTs with enhanced pathogen detection capabilities remains imperative. Importantly, more than 40 pathogens were detected solely by Probe-Capture Metagenomics, including all of the NTM, *T. whipplei*. Studies have shown that compared with HIV-uninfected patients, significantly higher burden of *T. whipplei* colonization and NTM infection in the lungs have been observed in HIV-infected patients (Tan et al., 2023; Lozupone et al., 2013). Therefore, Probe-Capture Metagenomics showed obvious advantages in the detection of NTM and *T. whipplei* compared with CMTs. However, pulmonary infection caused by *T. whipplei* is rarely reported in PLWH (Yan et al., 2021; Stein et al., 2013). Whether *T. whipplei* could cause pathological changes in the lung should be further explored. Other commonly pathogens, such as *Aspergillus*, *T. marneffei*, could also be detected with high sensitivity. Moreover, the results can be obtained within 24 h, which was shorter than multiple CMTs. These results suggested that Probe-Capture Metagenomics combines the advantages of the speed, unbiased, high sensitivity and specificity of mHTS to detect multiple pathogens simultaneously (Han et al., 2019). Therefore, Probe-Capture Metagenomics which represent a valuable adjunct to CMTs can be indicated in the following clinical scenarios: (1) when CMTs fail to identify suspected opportunistic pathogens, especially in immunocompromised hosts, atypical presentations, or situations requiring rapid diagnosis; (2) for suspected polymicrobial co-infections; and (3) when CMTs has limited coverage, such as for emerging viruses or atypical bacteria. Notably, multiple organisms were diagnosed by Probe-Capture Metagenomics or CMTs alone in our study, which showed that it is still impossible for Probe-Capture Metagenomics to completely replace CMTs in clinical application. And detection from Probe-Capture Metagenomics cannot be considered the same as confirming it as an infectious agent, just that its genetic material could be found in the samples. Thus, Probe-Capture Metagenomics alone cannot distinguish pathogens between colonization and infection.

Through our study, a deeper understanding of the microbial spectrums of pulmonary was gained in PLWH with pneumonia. *T. whipplei*, *E. faecium*, *K. pneumoniae*, *P. aeruginosa* and *S. pneumoniae* in bacteria spectrum, *P. jirovecii* and *C. albicans* in fungus spectrum, CMV and HHV-7 in virus spectrum, MTB and MAC in mycobacterium spectrum were more commonly detected among PLWH with pulmonary infection. These results were partly similar with the study of Tan et al., which also showed that MTB and MAC were more common in the mycobacterium spectrum (Tan et al., 2023). And the detection ratios of CMV and *P. jirovecii* in PLWH with CD4^+^T count < 200 cells/μL were significantly higher than that with CD4^+^T count ≥ 200 cells/μL, which indicated that the pathogen spectrum of pulmonary infection varies in PLWH according to their immune conditions (Xu et al., 2022; Tan et al., 2023). In addition, HIV or other RNA viruses such as the SARS-CoV-2 can also be detected by Probe-Capture Metagenomics, which was superior to conventional mHTS method mainly detecting DNA viruses. Therefore, our findings could help physicians early recognize the pathogens of pulmonary infection in PLWH, which facilitates rapid and correctly empirical treatment for favourable prognoses of opportunistic infections. Notably, the detection rates of SARS-CoV-2 and influenza virus were low in our study. Since the onset of Omicron, all variants are from the same lineage and one of these have a higher affinity against cells in the upper respiratory tract, so lung cells are secondary (contrary to Delta and earlier variants). While Delta relies on ACE2-TMPRSS2-mediated entry in pulmonary tissues, the Omicron variants do not induce cell syncytia *in vitro* and favoured a TMPRSS2-independent endosomal entry pathway (Meng et al., 2022; Willett et al., 2022).

Currently, although effective ART has significantly improved survival in PLWH (Poorolajal et al., 2016), it only control the HIV level in PWLH below detection threshold and can not achieve a complete cure for HIV (Ryom et al., 2022; Chun et al., 1997; Lu et al., 2018). One important reason is that ART cannot eliminate the latent HIV reservoir in multiple cellular at diverse anatomical sites which can undergo clonal expansion and persist for many years (Ryom et al., 2022; Chun et al., 1997; Lu et al., 2018). Therefore, a comprehensive understanding of HIV reservoirs and their kinetics would be crucial in formulating efficacious to achieve a cure for HIV infection. Some sequencing methods to measure and characterize the HIV reservoir are also gradually emerging (Moar et al., 2023). But one single approach cannot be universally used due to its own advantages and limitations. Nowadays, researchers have widely recognized that in tissues, potential HIV reservoir has also been found in seminal vesicles, the urethra, adipose, liver, lung, bone marrow in femoral head as well as the central nervous system (Chen et al., 2022). Researches have demonstrated that the lung also serves as an HIV reservoir. HIV primarily infects CD4^+^T cells, while also capable of infecting lung-resident immune cells such as alveolar macrophages and dendritic cells (Cribbs et al., 2020). HIV-related lung injury and disease can occur secondary to a number of mechanisms including altered pulmonary and systemic inflammatory pathways, viral persistence in the lung, oxidative stress with additive effects of smoke exposure, microbial translocation, and alterations in the lung and gut microbiome (Cribbs et al., 2020). Our results suggested that Probe-Capture Metagenomics can detect HIV in the BALF of PLWH with pneumonia, providing further evidence that the lungs act as an important HIV reservoir, and Probe-Capture Metagenomics provides a supplement detection method for finding reservoirs of latent HIV.

HIV drug resistance (HIVDR) will impair the efficacy of ART, leading to virologic failure, and hinder the progress in HIV/AIDS treatment (Zhu et al., 2024). HIVDR assays are usually performed through Sanger sequencing, which could detect the frequency of minority variants at a 15–20% in PWLH (Casadellà and Paredes, 2017). In recent years, studies have shown that HTS was able to detect more of the minority drug resistance variants than Sanger (Li et al., 2021). Furthermore, although serum HIV drug resistance testing (HIVDR) are common, HIVDR in BALF still holds greater clinical significance. Different resistance mutations can be detected simultaneously in the blood and the lung of HIV-1 infected individuals on antiretroviral therapy (White et al., 2004). HIV strains in the lung may evolve independently from those in blood, developing unique drug resistance mutation profiles (White et al., 2004; Shahid et al., 2022). Consequently, even when blood viral load is well-controlled, latent virus in the lungs may still lead to localized viral replication and treatment failure. In our study, we discovered DRMs for NNRTI in 3 patients by Probe-Capture Metagenomics, which was targeted towards the HIV genome. This suggested that Probe-Capture Metagenomics could be used as a valuable additional tool to gather further data on HIVDR. However, the detection rate was not high in our study. In the future, as the gene probes for HIV resistance increase in Probe-Capture Metagenomics, its application value will improve in PLWH.

Furthermore, the antimicrobial resistance genes were detected in the BALF of 27 patients through Probe-Capture Metagenomics. In one previous study, by the direct detection of key gene features associated with carbapenem resistance of *P. aeruginosa*, the carbapenem resistance of *P. aeruginosa* was directly predicted from cultured isolates by HTS, which could assist the timely treatment and surveillance of carbapenem-resistant *P. aeruginosa* (Liu et al., 2023). This demonstrated that Probe-Capture Metagenomics is capable of detecting antimicrobial resistance genes in bacterial strain and can offer improved guidance for clinical medication. But the results of Probe-Capture Metagenomics were not completely consistent with BALF bacterial culture. Thus, Probe-Capture Metagenomics cannot completely replace culture in terms of antimicrobial resistance genes detection. In the future, it is still necessary to further optimize the sensitivity of probes in Probe-Capture Metagenomics, and then improve the application value in the detection of antimicrobial resistance genes.

Some limitations also exist in our study. Firstly, while RPM normalization accounts for sequencing depth, it does not adjust for genome size or nucleic acid type, which could bias cross-species abundance comparisons. However, as our analysis focused on pathogen detection within patient samples using a fixed RPM threshold, these factors did not impact our conclusions. Secondly, Probe-Capture Metagenomics does not detect actual Virus-like particles or organisms but rather their nucleic acids. Thus, the organisms detected by Probe-Capture Metagenomics may not be actual pathogenic microorganisms. Therefore, it is crucial to remove any contaminating and/or colonizing bacteria, taking into consideration the patient’s clinical history and other results from CMTs. Thirdly, the duration of antibiotic therapy prior to sampling was not considered in our study. Future research is needed to investigate the impact of antibiotic usage duration on the pneumonia microorganisms in the unique vulnerable population of PLWH. Finally, our study was limited by a small sample size, which was confined to a single center. The reliability of Probe-Capture Metagenomics needs to be verified with a larger sample size. Thus, the impact of Probe-Capture Metagenomics on regime adjustment of anti-infection and ART needs further investigation.

## 5 Conclusion

Probe-Capture Metagenomics provides a promising method for detecting suspected opportunistic infections in PLWH with pneumonia, especially for mixed infections and rare microorganisms. And the lungs may be a reservoir for HIV. In addition, Probe-Capture Metagenomics was a potential valuable tool for detecting HIVDR and antimicrobial resistance genes in bacteria.

## Data Availability

The original contributions presented in the study are publicly available. This data can be found here: https://www.ncbi.nlm.nih.gov/, PRJNA1254612.

## Footnotes

^1^ https://www.hopkinsguides.com/hopkins/index/Johns_Hopkins_ABX_Guide/Pathogens

^2^ http://hivdb.stanford.edu

## Glossary

PLWH: people living with HIV
CMTs: conventional microbiological tests
mHTS: metagenomic high-throughput sequencing
BALF: Bronchoalveolar Lavage Fluid
G test: 1,3-*β*-D glucan (G) assay
GM: galactomannan
PCR: polymerase chain reaction
CMV: cytomegalovirus
*P. jirovecii*: *Pneumocystis jirovecii*
MTB: *Mycobacterium tuberculosis complex*
TST: tuberculin skin test
Xpert: GeneXpert MTB/RIF
ART: antiretroviral therapy
CRP: C-reactive protein
IL-6: Interleukin-6
PCT: procalcitonin
ESR: erythrocyte sedimentation rate
LDH: lactate dehydrogenase
ALT: alanine aminotransferase
NTC: non-template controls
RPM: reads per million
CRR: clinical reportable range
INSTI: integrase strand transfer inhibitor
RT: reverse-transcriptase inhibitor
PI: protease inhibitor
MDT: mutation detection threshold
ESBL: extended-spectrum β-lactamases
*C. albicans*: *Candida albicans*
*T. whipplei*: *Tropheryma whipplei*
*T. marneffei*: *Talaromyces marneffei*
MAC: *Mycobacterium avium* complex
NTM: nontuberculous mycobacteria
DRMs: drug-resistance mutations
NNRTI: nonnucleoside reverse transcriptase inhibitors
VOA: virus outgrowth assay
HIVDR: HIV drug resistance
