# Supplementary material for: Analysis of microorganisms and drug-resistance mutations detected by probe-capture metagenomics among HIV-infected patients with pneumonia
Source: Front Microbiol. 2025 Jul 28;16:1616937. doi: 10.3389/fmicb.2025.1616937 (PMC12336185; doi:10.3389/fmicb.2025.1616937)
Supplement: Supplementary file 2 [file Table_2.docx]

Comparison of diagnostic performance among HC-based HTS and CMTs in 91 PLWH with pulmonary infection

| **CMT** | **mNGS** | CMV | PJP | MTB | Candida albicans | Aspergillus |
| --- | --- | --- | --- | --- | --- | --- |
| negative | negative | 21 | 33 | 67 | 60 | 53 |
| negative | positive | 22 | 9 | 4 | 25 | 13 |
| positive | negative | 2 | 9 | 7 | 1 | 18 |
| positive | positive | 46 | 40 | 13 | 5 | 7 |
| McNemer | P | 0.000036 | 1 | 0.548828 | 8.05E-07 | 0.47313 |

Comparison of diagnostic performance among HC-based HTS and CMTs in patients with CD4^+^T count ＜ 200 cells/µL

| **CMT** | **mNGS** | CMV | PJP | 结核 | 白念 | 曲霉 |
| --- | --- | --- | --- | --- | --- | --- |
| negative | negative | 12 | 23 | 55 | 47 | 41 |
| negative | positive | 18 | 4 | 4 | 20 | 11 |
| positive | negative | 2 | 7 | 4 | 1 | 14 |
| positive | positive | 41 | 39 | 10 | 5 | 7 |
| McNemer | P | 0.0004 | 0.5488 | 1 | 0.0001 | 0.69 |
